# The contribution of family physicians to district health services in South Africa: A national position paper by the South African Academy of Family Physicians

**DOI:** 10.4102/safp.v64i1.5473

**Published:** 2022-03-17

**Authors:** South African Academy of Family Physicians

**Affiliations:** 1 South African Academy of Family Physicians, Cape Town, South Africa

**Keywords:** family physicians, human resources, policy, workforce, district health system, district health services

## Abstract

The purpose of this position paper by the South African Academy of Family Physicians (SAAFPs) is to inform decision making on human resources for health policy in South Africa and the placement of family physicians (FPs) in the district health system. National policies have been marred by misunderstanding of the roles and contribution of FPs; and there is unhelpful variability in how FPs are positioned in the health services between provinces. In the private sector, medical aid schemes have discriminated against FPs by failing to remunerate them as specialists and to recognise their scope of practice.

## Summary

Family physicians (FPs) should be employed in primary health care (PHC) services and district hospitals. The creation of district clinical specialist teams provides an opportunity for most of the districts to obtain an additional FP, but their deployment should not be limited to these teams. Family physicians are not intended to be employed at regional or tertiary hospitals or as clinical managers. Their key roles include that of a clinician and consultant, a capacity builder and clinical trainer and a leader of clinical governance to improve the quality of care and promote patient safety. Family physicians improve individual and population health outcomes through engagement in community-orientated primary care. Their strategic deployment is a cost-effective intervention to strengthen district health systems because they work as the most senior clinician in multidisciplinary, collaborative and team-based practices. Family physicians are well suited to manage the complexity of the system in the best interests of the patients.

South Africa must expand its FP workforce throughout the district health system to achieve improvements in provincial strategic plans that focus on clinical outcomes at PHC level. South Africa (SA) should aim for at least one FP in each district hospital and in each community health centre or sub-district (without a community health centre) by 2030. There needs to be a substantial increase in the number of registrars in training and the number of FP posts by 2030. The essential contribution of FPs to the district health system needs to be incorporated into policy on implementation of National Health Insurance. Medical aid schemes need to meet their obligations to remunerate FPs appropriately and recognise the scope of practice of FPs as specialists. Accreditation of hybrid primary care practices with a mix of specialists (FPs) and non-specialists (general practitioners) is essential in the private sector.

## Introduction

This position paper on family physicians (FPs) in SA puts forward the viewpoint of the South African Academy of Family Physicians (SAAFPs). The SAAFP was established in 1980 to represent family doctors with a focus on primary health care (PHC) and was re-orientated in 2007 to be the national professional body representing the new speciality of family medicine. The SAAFP has over 600 members across all provinces and represents SA in the World Organisation of Family Doctors (WONCA). This article is intended to guide national policymakers in their planning, by deepening their understanding of the contribution FPs make to the national health system. In particular, we hope that this article will inform the new human resources for health policy and the placement of FPs in the district health services.

Historically, South African health policy documents have exhibited an inconsistent understanding of the role of FPs within the healthcare workforce. The previous human resources for health policy wrongly classified family medicine as a sub-speciality of internal medicine.^1^ Such thinking was reiterated in the recent ministerial task team report that only considered the need for specialists in tertiary and quaternary hospitals.^2^ They therefore made recommendations for family medicine that were based on a misunderstanding of the roles of FPs within the health system. Likewise, in PHC they only considered the need for FPs in district clinical specialist teams (DCSTs).

A recent analysis of human resources for health reported 969 FPs on the Health Professions Council of South Africa (HPCSA) register in 2019.^3^ Most were grandfathered from the previous register, prior to full recognition of the speciality in 2007, with only 176 entering from the new full-time registrar training programmes by 2019. It is important to note that provincial health authorities have adopted diverse approaches to incorporating these specialist FPs into their health services.

Family physician specialists are concentrated in Gauteng, Western Cape and KwaZulu-Natal.^3^ The national Education and Training Committee of the SAAFP provides feedback on deployment of FPs in different provinces. In the Western Cape, FPs have been appointed at community health centres and small- to medium-sized rural and urban district hospitals. In Gauteng, they have been employed mainly by the districts with oversight and managerial roles, in addition to the provision of direct primary care health services at community health centres (CHCs), district hospitals and PHC clinics. In KwaZulu-Natal, FPs have been appointed mostly in large urban hospitals, although recently there has been an intention to appoint them at rural district hospitals. In other settings, a few posts have been created at regional and tertiary hospitals and not in the district health services. In the private sector, medical aids, with the exception of Discovery Health, have failed to recognise the specialist status of FPs and their scope of practice.

## Policy context

The health policy context in SA is defined by our national commitment to the Sustainable Development Goals and in particular Goal 3 on good health and well-being.^4^ Our commitment to Universal Health Coverage^5^ (UHC) follows closely on this, along with the Astana Declaration on PHC.^6^

The World Health Organization (WHO) has published an operational framework for PHC to turn this global vision into action, with three key levers^7^:

- Multisectoral policy and action
- Empowered people and communities
- Integrated health services with an emphasis on primary care and essential public health functions.

The WONCA has also signed a memorandum of understanding with the WHO to support the operational framework and the contribution of family doctors to it.^8^ In Africa, there has been increasing engagement between WONCA and the WHO around implementation of PHC and district health services.^9^

South Africa is a ‘trailblazer’ country for the measurement of PHC in the PHC Performance Initiative – showing its political commitment to improving PHC.^10^ South Africa has made a commitment to deliver on UHC through implementation of national health insurance (NHI),^11^ although implementation appears to be stalled by the coronavirus disease 2019 (COVID-19) pandemic. Strengthening district health services is also clearly articulated in the National Health Plan, where FPs are considered a key human resource for improving quality of care and patient safety.^12^

Currently SA has no human resources for health policy, which is a major problem. Such a policy is needed to guide both the higher education sector on training of the health workforce and the health sector on deployment of health professionals. The development of a new policy appears to be stalled by the attention needed for the COVID-19 pandemic and changes in leadership within the National Department of Health.

## Health services context

Whilst a comprehensive overview of the national health services context is beyond the scope of this position paper, a thumbnail sketch of the key issues is outlined as a backdrop to the SAAFP’s position on FPs.

South Africa suffers from a quadruple burden of diseases, including human immunodeficiency viruses (HIV) and tuberculosis (TB); chronic non-communicable diseases and mental health; injury and violence; and maternal, neonatal and child health.^13^ Challenges with tackling this burden include an absolute shortage of frontline healthcare practitioners, maldistribution of the health workforce (between public and private sectors and rural and urban areas), crumbling infrastructure, inadequate health information systems without electronic medical records, resource constraints and difficulties with medication stock-outs and other shortages of supplies in many areas.^4^ In short, there are gaps in many of the health system building blocks and essential inputs for effective service delivery.

Within the district health services, significant skills gap have been identified at small- and medium-sized district hospitals, both rural and urban, particularly in surgery, anaesthetics and obstetrics.^14,15,16^ In PHC, patients rated first contact accessibility, ongoing care and community orientation as the poorest performing elements; first contact utilisation, informational coordination and family-centredness as weaker elements; and comprehensiveness, coordination, cultural competency and availability of the PHC team as stronger aspects of primary care.^17^ Moving beyond the biomedical, a national PHC survey showed that mental and social health problems were mostly not recorded during consultations, indicating that our primary care lacks a ‘broad and holistic perspective to the patient’s problems’,^18^ which is a hallmark of medical generalism and family medicine.^19^ From a more positive perspective, the Ideal Clinic initiative defined a foundation for quality primary care through norms and standards and has assisted with incremental improvement in both facilities and services offered.^20^ South Africa’s HIV/AIDS programme has benefited from additional vertical funding, successfully delivering the world’s largest anti-retroviral programme, although there are challenges with integration of this programme into district health services.^21^

District clinical specialist teams have worked to improve maternal and child outcomes within a primary care framework, although not all districts have a full team complement. Ward-based PHC outreach teams have been introduced to improve community-orientation and population health management.^22^ Although widely implemented, the efficacy of these teams has been hampered by problems with governance, community health worker compensation, primary care facilities, community involvement, training and information systems.^23^ The need for more collaborative and effective low and middle level management and leadership has been widely reported.^24^

Whilst there has been laudable political commitment and policy formation around PHC and the district health services,^4^ this is accompanied by many shortfalls in implementation and service delivery scale up. Greater active engagement with FPs may assist in effective implementation of existing policies. The many proven benefits of PHC in terms of improved health status, responsiveness to people’s needs, efficiency, resilience and equity have yet to be fully realised in our country.^4,10^ Given the political will and policy frameworks, SA has huge potential to make substantial strides through enhancing PHC and district health services.

## Contribution of family physicians to the health system

Family physicians are trained to work clinically in PHC and district hospitals. In essence, FPs improve individual patient care and population health management.^25^ As specialists in family medicine, FPs contribute to the district health services through a number of roles that are outlined here. The competencies that underpin these roles and the ability to lead individual and community-orientated care in the district is what distinguishes this specialty from others.^26^

### Clinician and consultant

The FP is firstly a clinician who brings much needed clinical expertise across a wide range of health conditions to both the PHC team and the district hospital staff complement. Family physicians are the most senior clinicians within these multidisciplinary teams and act as medical consultants. Whilst all members of the team are needed to ensure coverage to the population, the FP ensures the comprehensiveness and quality of services delivered, whilst also assessing and managing complex patients who might otherwise be referred to a higher level of care. Examples include patients with multi drug-resistant TB and HIV or women with pregnancy-induced hypertension or adults with heart failure and other comorbidities. In district hospitals, FPs bridge skills gap, particularly strengthening surgical, anaesthetics and obstetric care capacity.^27^ They also provide a consultancy role in this context ensuring that the full package of comprehensive care is available at district hospitals and likewise reduce the need to transfer patients to the next level of care. Patients benefit from accessing comprehensive, quality care close to their homes.^28^

In PHC the clinical team consists of nurse practitioners, medical officers, professional and enrolled nurses and community health workers. The PHC team spans both the facility and the community served by that facility. In district hospitals, similarly, the clinical team consists of medical officers, clinical associates and nurses. In both settings, the extended team consists of other allied health professionals, pharmacists and support workers. The FP therefore adopts a collaborative and team-based approach to service delivery and adapts their contribution to the local context.

Family physicians are trained in 10 clinical domains to manage patients appropriately in district health services: general adult medicine; child health; maternal and women’s health; trauma and emergency medicine; HIV, TB and sexually transmitted infections; surgery; anaesthetics; orthopaedics; ear, nose and throat (ENT); skin problems; and mental health.^26^ In addition, they are trained to comprehensively intervene across the life course and provide healthcare from health promotion, disease prevention and treatment to rehabilitation and palliative care. Family physicians are thus able to offer clinical leadership across all key domains^29^: reproductive, maternal, neonatal and child health (RMNCH), childhood illness, infectious diseases, non-communicable diseases, mental health and addiction, trauma, emergency medicine and palliative care’.

Family physicians are also committed to the biopsychosocial approach and person-centred care. They strengthen whole person medicine and the ability of teams to offer person-centred care, taking into consideration the social determinants of health for patients and their communities.^30^

Family physicians are also trained in community-orientated primary care and the clinical support required by ward-based outreach teams (WBOTs).^31^ For example, they can perform a home visit in support of the WBOT or consult on patients seen in the community setting. They are the only clinical specialist explicitly trained in the community oriented primary care (COPC) approach, and therefore invaluable to the implementation of COPC and WBOTs.

### Capacity builder and clinical trainer

In both PHC and district hospital clinical teams, FPs envision themselves as capacity builders.^32^ This means that they are both a role model for person-centred clinical care to the rest of the team, a consultant who provides feedback and advice on clinical care and a trainer who provides continuing professional development opportunities. Primary care teams are often made up of relatively junior doctors (medical interns, community-service doctors or grade 1 medical officers) or practitioners with less pre-service training (professional nurses, post-basic PHC nurse practitioners, clinical associates or community health workers). Ongoing, experiential, adult learning and capacity building are therefore necessities for skills development and retention of staff. Family physicians are specifically trained in a set of skills appropriate to clinical training and teaching in the workplace.

The new 6-month rotation in family medicine for medical interns requires expertise in hands-on clinical training and teaching within the health services. The FP is the ideal person to deliver and coordinate such training. It should also be observed that training of clinical associates for district hospitals also falls under family medicine and the presence of a FP is essential.^33^ In addition, it is now possible for medical officers in PHC to upskill themselves via the Diploma in Family Medicine (offered by several universities and the College of FPs) and the presence of an FP will enable the training required.^34^

Family physicians are also required for the delivery of more formal training of under- and post-graduates who are students enrolled in university degree programmes. Registrars in family medicine, future FPs, need to be trained for 4 years under the supervision of a FP. Training should predominantly take place in the district health services, where they will eventually work. In addition, undergraduate training of new doctors has shifted substantially towards an extended training platform or distributed platform. This is because of increasing medical student numbers and the shift in medical education from sub-specialist and specialist training in tertiary hospitals towards more appropriate generalist training in the district, closer to communities. Again, FPs are required to coordinate and provide such onsite training.

### Leadership and clinical governance

Family physicians are trained in leadership across all their roles, specifically in terms of their own values, goals and expertise; the need to lead through collaborative teamwork and relationships and the need to influence the key levers of the health system.^35^ One of their more specific roles is to help lead the implementation of COPC and WBOTs.^31^ Not only do they assist with upholding the principles of COPC during implementation but they also help to connect teams with the rest of the health system, other stakeholders in the area and community engagement.

One critical area for leadership is that of clinical governance at the district hospital, primary care facility and within COPC. This means improving the quality of care, enhancing service delivery and ensuring patient safety.^35^ Family physicians, as observed in the National Development Plan,^12^ are ideally positioned to lead clinical governance initiatives. They are trained to develop and implement clinical guidelines, undertake risk management through morbidity and mortality reviews, achieve quality improvement through audits and feedback, conduct patient satisfaction surveys and operational research^36^ and analyse routinely collected data and health information.^35^ Capacity building and clinical training, as described earlier, are also key aspects of clinical governance.

Closely related to clinical governance is corporate governance, which is a managerial function, assumed by facility managers, chief executive officers, clinical and medical managers. The FP is usually part of the facility management team and influences managerial thinking and planning for the key inputs required for service delivery, specifically regarding medication and other supplies; equipment; infrastructure; health information systems; an adequate, competent and motivated workforce; funding and budgets. In addition, FPs often act as line managers for other members of the clinical team. Although they are not trained primarily to be managers, it is observed that in many facilities FPs are being appointed in these posts.

Lastly, FPs bring systems thinking to the team, in that they can implement innovation and respond to changing health needs.^37,38^ Family physicians are trained to improve sequential and parallel coordination of care, for example, between primary and secondary care. They are also trained to improve informational and relational continuity, for example, through bolstering health information systems or ensuring ongoing care with the same team of health professionals. Finally, they give attention to improving access to care by looking at the acceptability of care to patients, opening times or appointment systems. Having someone with the ability to innovate and think in terms of systems is critical to improving the quality-of-service delivery.

### Other roles and contributions in the public sector

The development of DCSTs has been an opportunity for FPs to be employed in most districts and in many cases the FP is the anchor point for the team.^4^ These FPs in DCSTs help other specialists to navigate the district health system and have made a useful contribution to improving service delivery, particularly for maternal and child healthcare.^39,40^ However, FPs need to be employed throughout the district health services and not just in DCSTs and it is worrying that a recent ministerial task team on human resources for health policy only considered the need for FPs within these teams.^2^

In some provinces, FPs have been employed at regional and even tertiary hospitals. In some cases, they have provided useful clinical services, for example, running the emergency centre or filling gaps in services, however this is not the intended role of FPs. Family physicians are well-trained generalists who should be employed in generalist settings within the district health services. In some metropolitan areas, large district hospitals operate more like regional hospitals, with specialist departments and FPs are also not the ideal health cadre in these settings. Family physicians should be employed at scale in the appropriate setting to maximise their contributions.

### Contribution to the private sector

In the private sector FPs contribute similarly in terms of clinical care, capacity building and clinical governance. However, district hospitals do not really exist in this sector and FPs usually work as general practitioners in private practice. These FPs bring their expertise and extended scope of practice into this setting and can offer higher quality and more comprehensive care, particularly for more complex patients. Family physicians may work in solo practice, in group practices or for private healthcare organisations. In the longer term, it is hoped that all general practitioners would have some postgraduate training in family medicine.

### The family physician’s roles in a complex health system

A district health system is complex, not just complicated. This distinction is important in characterising the value of the FP to the district system. Such a system is complex because it is adaptive (ever-changing) and can at times be unpredictable. Everything from the patient’s experience of disease to the availability of resources is changing and often in unexpected ways. Through the roles as described in the given sections , the FP is an essential resource by which to achieve optimal results from the complexity of a district health system. The interdependence and multiplicity of the roles are required to manage the similarly multifaceted and interdependent parts of the district health system.

## Implications for human resource planning

There is no global agreement on how to calculate the number of FPs needed per population. The number will depend on resources, supply of FPs and the design of the health system. In SA currently we have about 0.16 FPs per 10 000 population.^3^ Similar middle-income countries have a range from 0.2 per 10 000 in Brazil to 1.2 per 10 000 in China, although all of these countries seek to increase the number of FPs.^41^ The recent ministerial task team suggested a goal of 0.2 per 10 000 although, as mentioned, this was based on faulty assumptions.^2^ High income countries range between 4.3 and 12.0 FPs per 10 000.^41^ A goal of 3.0 per 10 000 has been suggested for middle-income countries,^41^ but remains somewhat arbitrary.

The SAAFPs have previously suggested a more pragmatic, practical medium-term goal (by 2030) of at least one FP at every district hospital and community health centres or sub-districts (without community health centres).^3^ Taking into account the current gap, throughput of registrars enrolled in training and supply of new FPs, their likely distribution and loss to migration, SA will need to triple its current output to achieve this target.^3^ Although placing one FP at every facility in the district health system is our initial goal, we note that medium- and large-sized district hospitals will require more than one FP to function optimally. For example, in medium-sized district hospitals two FPs are often needed to cover the hospital and support the PHC platform. In larger district hospitals three or even four FPs may be needed to cover adequately all the clinical areas.

Our recommendation, therefore, is that every family medicine training programme requires an annual intake of 15–20 new registrars (between 60 and 80 registrars in total over the 4-year training programme).^3^ Although the capacity (HPCSA post numbers) already exists to enable such training, the average intake currently is only six to seven registrars for each of the nine training programmes.^3^ At this rate, we will not be able to guarantee a sufficient supply of FPs for the public sector and ensure that the goal is met.

In addition, we calculated the current gap for FPs in the public sector as 400.^3^ Therefore, each province should aim to create a minimum of five new FP posts per year for the next 10 years to achieve the stated goal.^3^ These posts should be created at small- to medium-sized district hospitals, community health centres or within sub-districts (that do not have a health centre).

## Budgetary implications

Although no formal cost-effectiveness studies have been conducted in SA, local anecdotal and international evidence tells us that primary care physicians make health systems more cost-effective.^42^ Experience of deploying FPs in the health system in SA provides examples of how FPs are cost-effective in our own context:

- Family physicians reduce the number of referrals from district health services to regional and tertiary facilities. Care in district health services is less costly and avoids the cost of transport.^27,43,44^
- Family physicians make district health services more efficient by improving the quality of care available and reducing the number of repeat visits needed to make an accurate diagnosis.^27,28,43,44^
- Family physicians capacitate the team and create a learning environment within which resources are used more efficiently.^45^
- Family physicians improve coordination of care between multidisciplinary teams in the district as well as between the district and the next level of expertise at referral hospitals. Building relationships of trust between specialists leads to reduced duplication of investigations and the potential for more shared care to happen in the district health services with support from hospital-based specialists.^46^

## Implications for funding and national health insurance

The need for FPs at district hospitals, community health centres and sub-districts needs to be incorporated explicitly into plans for NHI.^11^ Contracting units for PHC should ensure that FPs are part of their plans and appropriate remuneration for a specialist is included. Currently, there is no clear provision for FPs in the envisaged teams, accreditation process or remuneration calculations.^47,48,49^

## Implications for private sector

Medical aid schemes need to revise their remuneration of FPs as specialists in family medicine. Family physicians are being required to register as specialists with the Board of Healthcare Funders, but this is not aligned with how medical aids are remunerating them. Family physicians may also work in group practices with other non-specialist general practitioners and this arrangement should also be supported in how such practices are registered. At present, such hybrid practices are not being accommodated by the registration system.

In addition, beyond remuneration, the scope of practice of FPs should also be recognised. After four years of postgraduate training, their expertise and competencies in multiple roles provides a skill set very different from medical doctors without such training. These challenges within the private sector may lead to legal challenges in the near future.

## Summary of key recommendations

In conclusion, the SAAFPs make the following key recommendations:

- National policy should consistently understand the roles of the FP as described in this position article. Family physicians are trained as clinicians, consultants, capacity builders, clinical trainers and leaders of clinical governance.
- Family physicians are trained to be deployed across the entire district health system at primary care facilities (e.g. community health centres, general practices), district hospitals, sub-districts and as members of DCSTs.
- It must be recognised that FPs have broad roles to play within the district and should not only be utilised as members of DCSTs.
- Family physicians are not trained to be clinical or district level managers or for regional and tertiary hospitals.
- South Africa should aim for one FP in each district hospital, community health centre or sub-district (without a community health centre) by 2030.
- The number of new registrars admitted to each of the nine family medicine training programmes should be increased to at least 15–20 per year (60–80 total over a 4-year period).
- Each province should create a minimum of five new FP posts per year over the next 10-years to achieve the medium-term goal.
- The essential contribution of FPs to PHC teams and district hospitals needs to be made explicit and incorporated into national policy on the implementation of NHI.
- Medical aid schemes must meet their obligations to remunerate private sector FPs appropriately and recognise their scope of practice as specialists in family medicine.
- The private sector needs to allow accreditation of hybrid practices with a mix of specialists (FPs) and non-specialists (general practitioners).
- Ongoing research at provincial and national level should be carried out to evaluate the FPs’ contributions and impact on patient care, health services and population health outcomes.

## Conclusion

Family physicians are essential to improving the quality-of-service delivery and ensuring patient safety across the entire district health system. South Africa must commit to expand the training and deployment of FPs at district hospitals, community health centres and sub-districts. We hope that this position paper from the SAAFP will influence the evolution of our national human resources for health policy and the planning of provincial departments of health for employment of FPs. This position paper also addresses the differential treatment of FPs in the private sector. The SAAFP proposes pragmatic, practical and medium-term goals for 2030 and these will need to be revised as health services evolve.
